# Knowledge of the health personnel involved in the fluoride varnish therapy programs of primary schools in Tehran, Iran

**DOI:** 10.1186/s12903-024-04390-8

**Published:** 2024-06-01

**Authors:** Mohammad Reza Khami, Ali Haghparast Ghomsheh, Hossein Hessari, Mohsen Shati

**Affiliations:** 1 Research Center for Caries Prevention, Dentistry Research Institute, Tehran University of Medical Sciences, P.O. Box 1417614411, Tehran, Iran; 2 Department of Community Oral Health, School of Dentistry, Tehran University of Medical Sciences, Tehran, 1439955934 Iran; 3 Mental Health Research Center (MHRC), School of Behavioral Sciences and Mental Health, Tehran Institute of Psychiatry, Iran University of Medical Sciences, Tehran, Iran; 4 Department of Epidemiology, School of public health, Iran University of Medical Sciences, Tehran, Iran

**Keywords:** Fluoride, Topical, Health promotion, School dentistry, Education, Medical, Continuing

## Abstract

**Introduction:**

The World Health Organization (WHO) places great importance on oral health promotion programs in schools, given that approximately one billion people worldwide are students. This demographic not only includes the students themselves, but also extends to school staff, their families, and the broader community, all of whom are interconnected. The objectives of this study were firstly to assess the knowledge of health personnel conducting fluoride varnish treatment (FVT) in schools, and secondly to solicit their views on the effectiveness of their training methods.

**Methods:**

Data was collected from health personnel involved in FVT in schools, supervised by medical universities in Tehran province, using a questionnaire. The questionnaire was divided into four sections: demographic information, methods of receiving FVT training, respondents’ knowledge regarding FVT, and opinions about the effectiveness of FVT training methods. The questionnaire was distributed via social media, phone conversations, and email. The collected data was analyzed using Mann-Whitney in SPSS Version 26. A regression model was also fitted to the data.

**Results:**

The present study included 403 participants. Among various educational methods, it was found that participation in previous workshops (*P* = 0.001) and FVT workshops (*P* = 0.013) was significantly correlated with a higher FVT knowledge score. Additionally, participation in previous oral health promotion programs was significantly associated with a higher knowledge score (*P* < 0.05). Therefore, a history of participating in previous health promotion programs significantly contributed to the participants’ knowledge.

**Conclusion:**

Participation in previous oral health programs was found to be significantly correlated with a higher knowledge score. The effectiveness of training programs can be attributed to participation in previous workshops and FVT workshops. This study provided insights into potential strategies for enhancing personnel training in national oral health programs.

## Introduction

Over 530 million children suffer from dental caries globally, with the vast majority being students or preschoolers [1, 2]. Overlooking the issue of dental caries can lead to an increase in living expenses, particularly the cost of treatment, and can place financial strain on a country’s insurance and public health funds. Dental caries and the ensuing toothache can considerably affect a child’s quality of life [3]. Research indicates that six-year-old Iranian children, who are of school age, typically have more than four decayed teeth [4].The incidence of caries in these children has risen to an average of four and a half per child [5]. According to a 2012 report from Iran’s Ministry of Health and Medical Education (MOHME), the average Decayed, Missing, and Filled Teeth (DMFT) score was 5.6for Iranian children. Of this, 3.3, or approximately 59.3%, was attributed to the decayed component. The average caries score for Iranian children was 5.16, with an onset rate of 86% in this age group. The same report indicated that the mean DMF score for 12-year-old Iranian children was 3.29, with a caries prevalence of 75% [6].

Schools are often viewed as crucial platforms for health promotion due to their educated and readily accessible populations. The WHO places significant emphasis on oral health promotion programs in schools, as approximately one billion people worldwide are students. This group, in addition to being a substantial demographic in itself, also has strong ties with school staff, their families, and the wider community [4]. Indeed, the educational potential among school children is substantial. Consequently, the WHO has identified school students as a key target group for oral health programs [3, 7].

The WHO has formulated a comprehensive plan for healthy schools. This plan is grounded in objectives, such as fostering a healthy school environment, promoting health education, providing health services within schools, offering food and nutrition services, encouraging sports and recreational activities, prioritizing well-being and mental health, ensuring employee health, and facilitating communication and collaboration with the broader community [3]. It can also lead to a reduction in inequality at the school level and ultimately in the society.

A variety of oral and dental health promotion programs have been implemented in Iran. In 1998, a tripartite agreement focusing on education, prevention, and treatment was signed between the MOHME and the Ministry of Education for school students aged 6–12 years. Subsequent to this, additional programs were initiated. For instance, one of the oral health promotion programs coordinated with child-related organizations to implement a plan aimed at improving the oral health of kindergarten teachers. Concurrently, preschool students underwent examinations and received fluoride varnish treatments (FVTs) [8, 9]. In 2014, a plan was initiated to utilize a mobile dental unit for delivering oral health promotion services to schools. The Student Oral Health Improvement Plan was designed around several key services: issuance of dental birth certificates, fluoride therapy, health education, and the provision of necessary treatments, including referrals when required [10].

In 2015, the most recent initiative, which was a new agreement for primary school oral health promotion, was signed between the MOHME and the Ministry of Education. It was named the Primary School Oral Health Promotion Program (PSOHPP), targeting children under 14 years of age. According to this program, services are provided free of charge for rural and less privileged areas, while in urban areas, parents cover 30% of the cost. Additionally, children receive free oral examinations. If a child is diagnosed with dental issues during these examinations, they are referred to government-funded dental centers, such as dental schools or clinics contracted by the Ministry of Education for treatment. During these student examinations, the examiner also spends a few minutes on oral health education [11]. Another component of this program is the administration of FVT twice a year.

The addition of FVT to caregiver counseling has proven effective in reducing the incidence of early childhood caries [12]. This approach has been implemented in school-based oral health programs globally [13]. The FVT is typically administered by a trained health professional who is knowledgeable about the procedure. A diverse group of these professionals, ranging from community health workers in rural areas to dental hygienists and dentists in urban locales, are employed by the MOHME to carry out FVT in the PSOHPP program. However, the majority of this personnel comprises non-dental professionals who have been specifically trained to administer FVT as part of this program. On the other hand, the success of the FVT program is highly dependent to the knowledge of its operators [14], as essential considerations should be taken into account when performing the procedure [15]. Evidently, evaluating the knowledge of these professionals and its correlation with their training methods can provide valuable insights for enhancing this program and informing future initiatives.

In Iran, the universities of medical sciences, which are supervised by the MOHME, are tasked with providing healthcare services to the populations under their jurisdiction. Tehran, with a population exceeding nine million, is served by three main universities of medical sciences. Each of these universities covers distinct geographical areas within the city.

Despite the eight-year operation of the PSOHPP, there is, to the best of our knowledge, no comprehensive evaluation of the program, including an assessment of the involved personnel’s knowledge. Therefore, the aim of this study was firstly to examine the knowledge of health personnel who administered FVT in Tehran schools, and secondly to solicit their views on the effectiveness of their training methods.

## Methods

The present study was approved by the Ethics Committee of the School of Dentistry, Tehran University of Medical Sciences, Tehran, Iran(Code: IR.TUMS.DENTISTRY.REC.1400.138). Due to the ethical considerations from one of the three medical universities in Tehran (Iran University of Medical Sciences), our study was conducted in the remaining two universities (Tehran University of Medical Sciences and Shahid Beheshti University of Medical Sciences), which collectively serve populations of 3,160,817 [16] and 5,745,828 [17], respectively, according to the latest statistics available. Of these populations, around 11.5% have been in the age range of 6–14 years, and were candidate for FVT at the beginning of PSOHPP [18]. In these two universities 434 professionals were involved in FVT in Tehran schools.

The target population of our study consisted of health personnel who were involved in administering FVT in schools, under the supervision of medical universities in Tehran province. The primary instrument for this research was a researcher-made questionnaire (See Appendix 1). This data collection tool was divided into four sections:

- ***Demographic and background information***: This section collected information on age, gender, level of education, and history of participation in previous oral health promotion programs.
- ***Methods of receiving FVT training***: In this section, the participants were asked about the FVT training methods they had participated in. All methods applied to the FVT service providers were listed as a checklist, and the participants could select as many options as applicable. The options were: FVT workshop, Professional development courses, Pamphlets and posters, Booklets, CD.
- ***Knowledge of the respondents regarding FVT***: This section covered the nine steps required for a successful FVT checklist from a previously validated questionnaire [19]. The respondents were asked to rate their agreement with the importance of each step on a scale of 0–5, resulting in a possible score range of 0–45.
- ***Opinions about the effectiveness of FVT training methods***: In this section, the participants were asked to identify which FVT training methods they found to be more effective than others. The options were the same as those for “Methods of receiving FVT training” section, mentioned above.

The questionnaire was in Farsi language. In order to include all available options for the questions and to validate the check lists, the questionnaire and its items were discussed in three separate group discussions, one with the experts of Oral Health Bureau (MOHME), and two with dental officers of the two universities. Necessary amendments were done according to the experts’ opinion [20].

### Data collection

The contact list of service providers, including their phone numbers, was procured from the health vice-chancellors of medical sciences universities. A link to the online questionnaire was distributed among these individuals via SMS or the WhatsApp social network. If the questionnaire remained incomplete after two weeks, a reminder was sent to the participants. The reminder process was repeated after one week.

The data collection started in January 2022, and lasted for six weeks.

### Statistical analysis

To describe the data, frequency, median, mean, and standard deviation (SD) were calculated. Given the lack of normality in the data, non-parametric methods, such as Mann-Whitney was employed for data analysis. A regression model was fitted to the data to explore the relationships of the independent variables to the participants’ knowledge of FVT. The gathered data was processed using SPSS Version 26.

## Results

Out of the 434 links distributed, a total of 403 participants (representing 93% of the recipients) took part in the study. The average age of the participants was 36.1 years, and they had an average of 5.93 years of work experience. Of these participants, 30 (7.44%) were men, and 375 were women. In terms of educational attainment, 223 (55.3%) of the 403 participants held a bachelor’s degree or higher. Regarding the employment status, 119 (29.5%) participants had permanent contracts, while 284 (70.5%) were temporarily hired for this program. Additionally, 255 (63.3%) respondents had previously participated in oral health promotion programs.

**Table 1:** Frequency of health professionals reporting scores 4 and 5 for each knowledge statement in the schools ’fluoride varnish treatment (FVT) programs in Tehran (*n* = 403)

Awareness questions	Frequency of true responses (scores 4 &5) (%)
The importance of tooth brushing before applying fluoride varnish	390 (96.8)
The importance of proper mixing of fluoride varnish components	380 (94.3)
The importance of correct posture for children during fluoride varnish application	393 (97.5)
The importance of drying all dental surfaces before applying fluoride varnish	379(94.0)
The importance of isolating teeth from oral fluoride for FVT	338 (83.9)
The importance of applying fluoride varnish for all dental surfaces	385 (95.5)
The importance of keeping oral fluids away from dental surfaces for a while after application	332 (82.4)
The importance of giving instructions after FVT	396 (98.3)
The need for discipline	394 (97.8)

Table 1 presents the frequency of participants reporting scores of 4 and 5 for each knowledge statement. “The importance of keeping oral fluids away from dental surfaces for a while after FVT” statement ranked the highest, followed by “The importance of isolating teeth from oral fluids for FVT”. “The importance of giving instructions after FVT” received the lowest ranking.

**Table 2:** Distribution of the mean knowledge scores based on background factors among health professionals involved in the schools ’fluoride varnish treatment (FVT) programs in Tehran (*n* = 403)

Variables		Frequency (%)	Mean knowledge score	SD	*P*-value*
Age (years)	21–35	215 (35.3)	41.31	3.32	0.011
	36–45	133 (33.0)	41.34	3.35	
	46–64	55 (13.6)	42.54	3.25	
Work experience (years)	1–5	192 (47.6)	41.58	3.36	0.539
	6–10	211 (52.4)	41.40	3.33	
Education	Bachelor’s degree and above	223 (55.3)	41.37	3.39	0.484
	Others	180 (44.7)	41.63	3.28	
Occupation	Nurse	100 (24.8)	41.26	3.56	0.409
	Dentist	33 (8.2)	41.67	3.23	
	Health care worker /technician	47 (11.7)	42.61	2.48	
	Healthcare worker	46 (11.4)	42.06	2.96	
	Others	177 (43.9)	41.10	3.53	
Type of employment	Official staff	119 (29.5)	41.56	3.43	0.758
	Temporary employees	284 (70.5)	41.45	3.31	
Participation in previous oral health promotion programs	Yes	255 (63.3)	41.90	3.21	0.001
	No	148 (36.7)	40.76	3.44	

* Mann-Whitney test

Table 2 presents the average knowledge scores of the participants based on their background characteristics. A significant increase in the mean knowledge score was observed with advancing age (*P* < 0.05). Furthermore, participants who had previously engaged in oral health programs demonstrated a higher mean knowledge score compared to those without such experience (*P* = 0.001).

**Table 3:** Description of the status of using different educational methods by health professionals involved in the schools ’fluoride varnish treatment (FVT) programs in Tehran (*n* = 403)

Training method	Category	Frequency (%)	Mean knowledge score	SD	*P*-value*
FVT workshop	Yes	206 (51.1)	41.97	2.96	0.013
	No	197 (48.9)	40.97	3.63	
Professional development courses	Yes	227 (56.3)	41.36	3.50	0.701
	No	176 (43.7)	41.64	3.13	
Pamphlets and posters	Use	172 (42.7)	41.41	3.31	0.687
	Non-use	231 (57.3)	41.54	3.37	
Booklets	Use	239 (59.3)	41.61	3.21	0.595
	Non-use	164 (40.7)	41.31	3.54	
CD	Use	142 (35.2)	41.67	3.01	0.753
	Non-use	261 (64.8)	41.38	3.51	

*Mann-Whitney test

Table 3 describes the average knowledge scores of the participants, categorized by the methods through which they received FVT training (all of the participants had received education on VFT via at least one of the training methods). Participation in FVT workshops (*P* = 0.013) was significantly related to a higher FVT knowledge score.

**Table 4:** Description of the respondents’ opinion regarding the effectiveness of various educational methods and its relationship with their mean knowledge score of fluoride varnish treatment (FVT) (*n* = 403)

Training method	Effectiveness	Frequency (%)	Mean knowledge score	SD	*P*-value*
FVT workshop	Yes	158 (76.7)	41.98	3.08	0.081
	No	48 (23.3)	41.94	2.59	
Professional development courses	Yes	156 (69.0)	41.21	3.57	0.420
	No	71 (31.0)	41.70	3.35	
Pamphlets and posters	Yes	83 (48.3)	41.43	3.55	0.587
	No	89 (51.7)	41.39	3.08	
Booklets	Yes	135 (56.5)	41.62	2.97	0.449
	No	104(43.5)	41.58	3.38	
CD	Yes	67 (47.2)	41.61	3.29	0.735
	No	75 (52.8)	41.70	2.75	

* Mann-Whitney test

Based on the findings, no significant relationship was found between the opinions of the respondents regarding the effectiveness of various educational methods and their knowledge regarding FVT (Table 4).

**Table 5:** Relationship of FVT knowledge with the investigated factors among the healthcare personnel engaged in primary schools ’fluoride varnish treatment (FVT) programs in Tehran (*n* = 403) according to a regression model

Model	B	SE	*P*-value	95%CI
Intercept	40.839	0.462	< 0.001	(39.93, 41.75)
Age	0.350	0.230	0.129	(-0.10, 0.80)
History of attending FV Training workshops(Reference group: Not attended)	0.785	0.422	0.064	(-0.04, 1.61)
History of participation in previous oral health promotion programs (before FVT) (Reference group: Participated)	-0.981	0.343	0.004	(-1.65, -0.31)
Believing in effectiveness of FV Training workshop (Reference group: No)	0.097	0.423	0.818	(-0.73, 0.93)

Table 5 presents the regression model based on variables that exhibited a significant correlation with the knowledge score in prior tests. Notably, participation in earlier oral health promotion programs (before FVT) was significantly linked to a higher knowledge score (*P* < 0.05). This suggests that the knowledge score was independent of age and associated with the history of participation in previous health promotion programs. The knowledge score of individuals who attended previous FV Training workshops was nearly one unit greater than the score of those who did not participate in these workshops.

## Discussion

The objective of this study was to assess the knowledge of health personnel conducting FVT in schools of Iran and to determine its determinants. The results revealed that the overall knowledge of the participants was good. The significant presence of healthcare workers (“Behvarzes” in the Iranian medical system) in this study can be attributed to their status as the primary health workforce in the country’s rural areas, where they perform oral health tasks, such as oral examinations, FVT, and referrals [21]. Over 90% of the participants acknowledged the importance of executing the necessary steps for FVT, with the exception of two items related to tooth isolation; approximately 20% of the participants did not fully agree with these two items. It is worth noting that isolation is a critical step in FVT [22]; therefore, future programs should place more emphasis on it.

In the bivariate analysis, individuals of higher ages and those with prior experience in oral health programs reported significantly better knowledge. However, in the regression model, after adjusting for variables, only the correlation with previous experience remained significant, suggesting that the age-related difference is likely a reflection of experience. This underscores the importance of experience, indicating that these individuals will gain more knowledge if they continue with this program or participate in future health promotion programs. Involvement in previous oral health promotion programs provides insight about effectiveness of strategies, challenges, and the ways to improve outcomes [23, 24]. This insight increases the motivation to continue involvement in oral health programs, which, in turn, improves the success of these programs. For example, according to a study in the US, pediatricians reported “personal experience implementing oral health into their practice” as one of the main factors enabling their success in a new oral health initiative. This experience provided more interest and motivation for them to actively participate in the new program [24].

Among the various training methods, the FVT workshop was identified as the most effective. This observation aligns with the findings of previous studies involving non-health professionals, where it was found that training workshops enhanced the oral health knowledge of school teachers [25, 26]. Although interactive workshops could improve knowledge and participant satisfaction, combining interactive workshops with other teaching methods may be more successful [27].

Based on our findings, among the various educational methods and materials, CDs were reported to be the least utilized. Conversely, booklets were identified as the most frequently used training method. Generally, booklets are more accessible [28] and can be read without the need for a computer. Furthermore, a study in Iran showed that combination of booklet, pamphlet and Continuing Medical Education (CME) could significantly improve oral health knowledge for primary care physicians [29]. CME is the professional development courses specifically designed for those in the medical field [30].

Professional development courses are routinely offered to healthcare professionals to enhance the necessary skills for their profession; some of these courses require mandatory participation. Furthermore, these professionals can expedite their career progression by attending these courses. This is why professional development courses were ranked as the second most popular training method by the participants. These courses offer valuable capabilities for oral health promotion programs [29] and should be given greater consideration in future programs. Moreover, online platforms exist for these courses, enhancing their accessibility and popularity. Given the advancements in distance and digital education systems and the increased ease of access to these platforms, particularly in the wake of the corona virus pandemic, these methods could be incorporated into future programs. This could include hosting webinars or online classes and utilizing digital education tools and content, with a particular emphasis on leveraging artificial intelligence. On the other hand concerns exist about online CME, including technological limitations and lack of sufficient engagement of the participants [31].

One of the strengths of this study was its census-style approach, which ensured a satisfactory sample size of participants. However, our inability to directly observe FVT in schools due to the COVID-19 pandemic could have led to invalid results. The cross-sectional design of the study can be seen as another limitation. Our questionnaire was mainly in the format of checklist, extracted partly from a previously validated questionnaire [19]. We had no complex variable to go through routine validation process. However, we used experts’ opinion to validate checklist [20]. The authors suggest conducting separate studies to compare the success or potential failure of PSOHPP, as well as to gauge the satisfaction levels of project stakeholders in different regions of the country.

## Conclusion

According to the findings of this study, participation in previous oral health programs was significantly linked to a higher knowledge score. Moreover, engagement in prior workshops and FVT workshops can be viewed as an effective training method, both for the continuation of the current program and for future initiatives. This study could provide insights into how to enhance personnel training in national oral health programs.

## Data Availability

All data are available upon request. For more information please contact to correspondence email at: ahgh90382@gmail.com.
